# Supplementary material for: Translation initiation factor eIF1.2 promotes Toxoplasma stage conversion by regulating levels of key differentiation factors
Source: Nat Commun. 2024 May 23;15:4385. doi: 10.1038/s41467-024-48685-4 (PMC11116398; doi:10.1038/s41467-024-48685-4)
Supplement: Supplementary file 3 — Description of Additional Supplementary Files [file 41467_2024_48685_MOESM3_ESM.pdf]

Description of Additional Supplementary Files.

File name: Supplementary Data 1

Description: Mutations found within the coding regions of the 8 independent clones

1a. Mutations in the coding regions of Clone 5E4.

1b. Mutations in the coding regions of Clone 8E7.

1c. Mutations in the coding regions of Clone 1D10.

1d. Mutations in the coding regions of Clone 1E9.

1e. Mutations in the coding regions of Clone 1B8.

1f. Mutations in the coding regions of Clone 2H11.

1g. Mutations in the coding regions of Clone 5G9.

1h. Mutations in the coding regions of Clone 5G11.

File name: Supplementary Data 2

Description: Differential gene expression and differential translational efficiency analyses for total RNA sequencing (RNA) and ribosome profiling (Ribo) of ME49Δ*ku80* (WT) and ME49Δ*ku80*Δ*eif1.2* parasites after 24 h under unstressed or alkaline-stressed conditions, as related to Fig. 5 and Supplementary Figs. 8 and 9.

2a. Differential gene expression between unstressed Δ*eif1.2* and unstressed WT parasites.

2b. Differential gene expression between stressed Δ*eif1.2* and stressed WT parasites.

2c. Differential gene expression between stressed and unstressed WT parasites.

2d. Differential gene expression between stressed and unstressed Δ*eif1.2* parasites.

2e. Differential translational efficiency between unstressed Δ*eif1.2* and unstressed WT parasites.

2f. Differences in translational efficiency between stressed and unstressed WT parasites.

2g. Differences in translational efficiency between stressed and unstressed Δeif1.2 parasites.

2a-d, the P_{adj} values were derived using DESeq2, employing two-sided tests with adjustments for multiple comparisons. **2e-g**, the P_{adj} values were derived using DeSeq2 and edgeR in Riborex, employing two-sided tests with adjustment for multiple comparisons.

File name: Supplementary Data 3

Description: Primers, gRNAs, repair templates, antibodies, other staining reagents, and cross-referenced genes used in this study.

3a. Primers for qPCR and qRT-PCR.

3b. Primers used for cloning and genotyping.

3d. Primary, secondary antibodies and other staining reagents.

3e. Genes exhibiting a fold change of ⩾ 5-fold in chronic versus acute infection (*P* value <0.05 and *Q* value <0.05; *Pittman et al*.).

3f. Genes exhibiting a fold change of ⩾ 5-fold in acute vs chronic infection (*P* value <0.05 and *Q* value <0.05; *Pittman et al*.).

3g. Genes exhibiting a fold change of ⩾ 5-fold between tissue cysts and in vitro tachyzoites of *T. gondii* (P_{adj} < 0.05; *Ramakrishnan et al*.).

3h. Genes exhibiting a fold change of ⩾ 5-fold between in vitro tachyzoites and tissue cysts of *T. gondii* (P_{adj} < 0.05; *Ramakrishnan et al*.).

3e,f. According to *Pittman et al.,* the *P* and *Q* values were derived using Cuffdiff, employing one-sided tests with adjustments for multiple comparisons. **3g,h**. According to *Ramakrishnan et al*., the P_{adj} values were derived using DESeq2, employing two-sided tests with adjustments for multiple comparisons.
