# Supplementary material for: Translation initiation factor eIF1.2 promotes Toxoplasma stage conversion by regulating levels of key differentiation factors
Source: Nat Commun. 2024 May 23;15:4385. doi: 10.1038/s41467-024-48685-4 (PMC11116398; doi:10.1038/s41467-024-48685-4)
Supplement: Supplementary file 7 — Reporting Summary [file 41467_2024_48685_MOESM7_ESM.pdf]

nature portfolio

Corresponding author(s): Vernon B. Carruthers
Last updated by author(s): Apr 15, 2024

Reporting Summary

Nature Portfolio wishes to improve the reproducibility of the work that we publish. This form provides structure for consistency and transparency in reporting. For further information on Nature Portfolio policies, see our Editorial Policies and the Editorial Policy Checklist.

Please do not complete any field with "not applicable" or n/a. Refer to the help text for what text to use if an item is not relevant to your study.
For final submission: please carefully check your responses for accuracy; you will not be able to make changes later.

Statistics

For all statistical analyses, confirm that the following items are present in the figure legend, table legend, main text, or Methods section.

n/a	Confirmed	
☐	☒	The exact sample size (n) for each experimental group/condition, given as a discrete number and unit of measurement
☐	☒	A statement on whether measurements were taken from distinct samples or whether the same sample was measured repeatedly
☐	☒	The statistical test(s) used AND whether they are one- or two-sided<br>*Only common tests should be described solely by name; describe more complex techniques in the Methods section.*
☐	☒	A description of all covariates tested
☐	☒	A description of any assumptions or corrections, such as tests of normality and adjustment for multiple comparisons
☐	☒	A full description of the statistical parameters including central tendency (e.g. means) or other basic estimates (e.g. regression coefficient) AND variation (e.g. standard deviation) or associated estimates of uncertainty (e.g. confidence intervals)
☐	☒	For null hypothesis testing, the test statistic (e.g. F, t, r) with confidence intervals, effect sizes, degrees of freedom and P value noted<br>*Give P values as exact values whenever suitable.*
☒	☐	For Bayesian analysis, information on the choice of priors and Markov chain Monte Carlo settings
☒	☐	For hierarchical and complex designs, identification of the appropriate level for tests and full reporting of outcomes
☒	☐	Estimates of effect sizes (e.g. Cohen's d, Pearson's r), indicating how they were calculated

Our web collection on statistics for biologists contains articles on many of the points above.

Software and code

Policy information about availability of computer code

Data collection

Image Lab™ (v6.0.1) software was used for Molecular Imager® Gel Doc™ XR System (Bio-Rad).
Zen blue edition (v2.6) software was used for Zeiss Axio Observer Z1 inverted microscope.
BDFACSDiVa™ (v9.0) software was used for BD LSR Fortessa flow cytometer.
CFX Manager (v3.1) software was used for Bio-Rad CFX96 Touch Real-Time PCR Detection System.
Summit (v.6.3.1.16945) software was used for MoFlo Astrios cell sorter (Beckman Coulter).
Living Image® (v4.0) software was used for the IVIS® Spectrum in vivo imaging system (PerkinElmer).
Genesys (v1.8.2.0) software was used for Syngene PXi6 imaging system.
ImageQuantTL (v10.2.499) software was used for Typhoon imager.
Proprietary Pacbio software was used for Pacific Biosciences RS II.
Wolfram Mathematica (v13.1) software was used to run simulation.
MATLAB (v2017a) software was used for processing data from the single-molecule scanning assay.
Flowcell (v2.00T) software was used for the Gradient Master 108 (Biocomp).
BCL Convert (v4.2.4) software was used for both Illumina MiSeq and Illumina NovaSeq S4 in whole genome sequencing experiments.
Bcl2Fastq (v2.17) software was used for Illumina HiSeq 4000 for RNA-seq and Ribo-seq.

Data analysis

Fiji (v2.9.0/1.53t or v2.3.0/1.53q) software was used for quantifying Western blotting and immunofluorescence staining.
Flowjo (v10.10.0) software (BD Biosciences) was used for analyzing flow cytometry results.
Protein sequences were aligned using ClustalW in MEGA11(v11.0.13) or in MegAlign Pro (v17.5.0, DNASTAR). The visualization of phylogenetic tree was generated by ITOL (v6).
Statistical analyses were conducted using Prism (v10.2.0) or Rstudio (v2023.06.01+524) with the lme4, lm and emmeans packages.
SNP analysis was performed using the variant calling workflow (default settings) provided by VEuPathDB Galaxy Site (https://

veupathdbprod.globusgenomics.org/).
For RNA-seq and Ribo-seq data analysis, trimming was performed using Cutadapt (v3.7), followed by removal of rRNA and tRNA reads using Bowtie2 (v2.4.2). Alignment was conducted using STAR (v2.7.8a), and gene counts were obtained using Htseq-count (v2.0.5). Differential expression was carried out with DESeq2 (v2.11.40.8), while translational efficiency was assessed using RiboRex (v2.4.0).

For manuscripts utilizing custom algorithms or software that are central to the research but not yet described in published literature, software must be made available to editors and reviewers. We strongly encourage code deposition in a community repository (e.g. GitHub). See the Nature Portfolio guidelines for submitting code & software for further information.

Data

Policy information about availability of data

All manuscripts must include a data availability statement. This statement should provide the following information, where applicable:

- Accession codes, unique identifiers, or web links for publicly available datasets
- A description of any restrictions on data availability
- For clinical datasets or third party data, please ensure that the statement adheres to our policy

Unprocessed data from whole genome sequencing have been submitted to the Sequence Read Archive (SRA) database under the following accession number: PRJNA1033315.
Unprocessed data from the RNA-seq and ribosome profiling are available through Gene Expression Omnibus (GEO) under the following accession number: GSE245775.
All remaining data associated with this study are provided within this manuscript, its supplementary files, and the accompanying source data files.
The database utilized was ToxoDB (https://toxodb.org/toxo/app). The dataset from Ramakrishnan et al. was sourced from ToxoDB (https://toxodb.org/toxo/app/search/transcript/GenesByRNASeqtgonME49_Ramakrishnan_enteroepithelial_stages_ebi_rnaSeq_RSRCDESeq), while the dataset from Pittman et al. was acquired directly from their manuscript.

Research involving human participants, their data, or biological material

Policy information about studies with human participants or human data. See also policy information about sex, gender (identity/presentation), and sexual orientation and race, ethnicity and racism.

Reporting on sex and gender	N/A
Reporting on race, ethnicity, or other socially relevant groupings	N/A
Population characteristics	N/A
Recruitment	N/A
Ethics oversight	N/A

Note that full information on the approval of the study protocol must also be provided in the manuscript.

Field-specific reporting

Please select the one below that is the best fit for your research. If you are not sure, read the appropriate sections before making your selection.

☒ Life sciences ☐ Behavioural & social sciences ☐ Ecological, evolutionary & environmental sciences

For a reference copy of the document with all sections, see nature.com/documents/nr-reporting-summary-flat.pdf

Life sciences study design

All studies must disclose on these points even when the disclosure is negative.

Sample size	Sample sizes for the mouse experiments were determined based on preliminary experiments and similar studies from our lab (Di Cristina M. et al., Nature Microbiology, 2017; Smith D. et al., eLife, 2021). For other experiments, sample sizes were selected to ensure they provided sufficient statistical power for comparison between different groups. There were no predetermined sample sizes based on statistical methods. The specific sample sizes for each experiment are listed in either figure legends or the method sections.
Data exclusions	No data were excluded from this manuscript.
Replication	Each round of flow sorting experiments and whole genome sequencing of mutant clones constituted one replicate. Single-molecule scanning and polysome profiling experiments were conducted as two biological replicates. The remaining experiments were performed with a minimum of three biological replicates each. All attempts at replication were successful, and the number of replication attempts is detailed in the manuscript.

Randomization	Prior to infection with parasites of different genotypes, female mice of similar ages were randomly assigned to experimental groups. However, for other experiments conducted solely on parasites within human foreskin fibroblast, randomization was deemed unnecessary as all samples belonged to specific genotypes.
Blinding	Blinding procedures were implemented during the assessment of in vitro and in vivo cyst numbers to prevent bias, with investigators being unaware of the genotypes.

Reporting for specific materials, systems and methods

We require information from authors about some types of materials, experimental systems and methods used in many studies. Here, indicate whether each material, system or method listed is relevant to your study. If you are not sure if a list item applies to your research, read the appropriate section before selecting a response.

Materials & experimental systems

n/a	Involved in the study	
☐	☒	Antibodies
☐	☒	Eukaryotic cell lines
☒	☐	Palaeontology and archaeology
☐	☒	Animals and other organisms
☒	☐	Clinical data
☒	☐	Dual use research of concern
☒	☐	Plants

Methods

n/a	Involved in the study	
☒	☐	ChIP-seq
☐	☒	Flow cytometry
☒	☐	MRI-based neuroimaging

Antibodies

Antibodies used	Primary antibodies used in this study include rabbit anti-BAG1 (Carruthers Lab, University of Michigan), rat anti-SAG1 (Carruthers Lab, Univeristy of Michigan), rabbit anti-TgActin (Sibley lab, Washington University in St. Louis), mouse anti-TgActin (Soldati-Favre Lab, University of Geneva), mouse anti-Ty (Clone BB2, Sibley lab, Washington University in St. Louis), rat anti-HA (Clone 3F10, Roche, Cat#11867423001).<br><br>Secondary antibodies used in this study include goat anti-rabbit Alexa Fluor 594 (Invitrogen, Cat#A11012), goat anti-Rabbit IgG (H+L) Secondary Antibody, HRP (Fisher Scientific, Cat#32460), Peroxidase AffiniPure™ Goat Anti-Rat IgG (H+L) (Jackson ImmunoResearch Laboratories, Cat#112-035-143), Peroxidase AffiniPure™ Goat Anti-Mouse IgG (H+L) (Jackson ImmunoResearch Laboratories, Cat#115-035-146).
Validation	The rabbit anti-BAG1 antibodies have been previously successfully utilized in peer-reviewed articles (DOI: 10.7554/eLife.59384; DOI: 10.1128/mBio.01324-19).<br>The rat anti-SAG1 antibodies have been previously successfully utilized in a peer-reviewed article (DOI: 10.7554/eLife.59384).<br>The rabbit anti-TgActin antibodies have been previously successfully utilized in a peer-reviewed article (DOI: 10.1002/(SICI)1097-0169(1997)37:3<253::AID-CM7>3.0.CO;2-7).<br>The mouse anti-TgActin antibodies have been previously successfully utilized in peer-reviewed articles (DOI: 10.1038/s41467-022-27996-4; DOI: 10.1038/s41467-021-24083-y).<br>The mouse anti-Ty antibodies (BB2) have been previously successfully utilized in peer-reviewed articles (DOI: 10.1016/0166-6851(96)02598-4; DOI: 10.1016/j.cell.2019.12.013).<br>The rat anti-HA antibodies have been validated by the manufacturer for use in western blots (https://www.sigmaaldrich.com/US/en/product/roche/roahaha). These antibodies have been previously successfully utilized in peer-reviewed articles (DOI: 10.7554/eLife.39887).<br><br>All secondary antibodies have been validated by the manufacturer for use in western blots and/or immunofluorescence.

Eukaryotic cell lines

Policy information about cell lines and Sex and Gender in Research

Cell line source(s)	We obtained human foreskin fibroblasts (HFFs) from the American Type Culture Collection (ATCC) with catalog number ATCC-CRL-1634 (HS27).
Authentication	The cell line was not authenticated.
Mycoplasma contamination	Human foreskin fibroblast cells have been confirmed to be free of mycoplasma contamination through regular testing.
Commonly misidentified lines<br>(See ICLAC register)	The study did not involve the use of commonly misidentified cell lines.

Animals and other research organisms

Policy information about studies involving animals; ARRIVE guidelines recommended for reporting animal research, and Sex and Gender in Research

Laboratory animals	Albino and non-albino female C57BL6 mice aged 6 to 8 weeks , along with 7 to 8-week-old CBA/J female mice, were used in this study. These mice were obtained from Jackson Laboratories.
Wild animals	No wild animals were used in this study.
Reporting on sex	Given the comparable outcomes of acute and chronic infections with ME49 strain parasites in both male and female CBA/J mice (DOI: 10.1128/IAI.00024-19), we exclusively utilized female mice in our experiments to minimize unnecessary use of animals.
Field-collected samples	This study did not use field-collected samples.
Ethics oversight	All experiments involving mice were reviewed and approved by the Institutional Animal Care and Use Committee at the University of Michigan.

Note that full information on the approval of the study protocol must also be provided in the manuscript.

Plants

Seed stocks	N/A
Novel plant genotypes	N/A
Authentication	N/A

Flow Cytometry

Plots

Confirm that:

☒ The axis labels state the marker and fluorochrome used (e.g. CD4-FITC).

☒ The axis scales are clearly visible. Include numbers along axes only for bottom left plot of group (a 'group' is an analysis of identical markers).

☒ All plots are contour plots with outliers or pseudocolor plots.

☒ A numerical value for number of cells or percentage (with statistics) is provided.

Methodology

Sample preparation	For sorting, bradyzoites were liberated from host cells through scraping, syringe-lysis (using 20- and 25-gauge needles), pepsin treatment, filtration, and resuspension in PBS. For analysis, bradyzoites were harvested through scraping, syringe-lysis (using 20- and 25-gauge needles), pepsin treatment, and filtration. Purified parasites were then fixed with 4% formaldehyde for 15 minutes at room temperature, washed once with PBS, and resuspended in PBS for analysis.
Instrument	LSR Fortessa flow cytometer (BD Biosciences) and MoFlo Astrios cell sorter (Beckman Coulter) were used in this study.
Software	During data collection with the BD LSR Fortessa flow cytometer, we utilized BDFACSDiVa™ software (BD Biosciences). For data collection with the MoFlo Astrios cell sorter (Beckman Coulter), we used the Summit software (v.6.3.1.16945). Subsequently, flow results were analyzed using Flowjo software (v10.10.0) from BD Biosciences.
Cell population abundance	We sorted mutants of the same cell type to enrich those with higher tdTomato-ATG8 signal. Through flow analysis and whole genome sequencing, we successfully enriched independent mutants, each with distinct mutations, showing increased tdTomato-ATG8 signal by flow analysis. This confirms the effectiveness of sorting gating.
Gating strategy	Gating relies on fluorescence signals for GFP (FITC) or tdTomato (PE (561)). Figures demonstrating the gating strategy to define the boundary between FITC-negative and FITC-positive, as well as between PE(561)-negative and PE(561)-positive are presented in Figures 1,2 and Extended Data Figure.2.

☒ Tick this box to confirm that a figure exemplifying the gating strategy is provided in the Supplementary Information.

This checklist template is licensed under a Creative Commons Attribution 4.0 International License, which permits use, sharing, adaptation, distribution and reproduction in any medium or format, as long as you give appropriate credit to the original author(s) and the source, provide a link to the Creative Commons license, and indicate if changes were made. The images or other third party material in this article are included in the article's Creative Commons license, unless indicated otherwise in a credit line to the material. If material is not included in the article's Creative Commons license and your intended use is not permitted by statutory regulation or exceeds the permitted use, you will need to obtain permission directly from the copyright holder. To view a copy of this license, visit http://creativecommons.org/licenses/by/4.0/
